# The neuroregenerative effects of topical decorin on the injured mouse cornea

**DOI:** 10.1186/s12974-020-01812-6

**Published:** 2020-05-04

**Authors:** Mengliang Wu, Laura E. Downie, Liam M. Grover, Richard J. A. Moakes, Saaeha Rauz, Ann Logan, Haihan Jiao, Lisa J. Hill, Holly R. Chinnery

**Affiliations:** 1 Department of Optometry and Vision Sciences, The University of Melbourne, Parkville, Victoria 3053 Australia; 2 School of Chemical Engineering, University of Birmingham, Birmingham, B15 2TT UK; 3 Academic Unit of Ophthalmology, Institute of Inflammation and Ageing, Birmingham and Midland Eye Centre, Birmingham, UK; 4 Neuroscience and Ophthalmology Research Group, Institute of Inflammation and Ageing, University of Birmingham, Birmingham, B15 2TT UK; 5 School of Biomedical Sciences, Institute of Clinical Sciences, University of Birmingham, Birmingham, B15 2TT UK

**Keywords:** Corneal sensory nerves, Nerve regeneration, Decorin, Dendritic cells, Macrophages, Immunomodulation

## Abstract

**Background:**

The cornea is innervated with a rich supply of sensory nerves that play important roles in ocular surface health. Any injury or pathology of the corneal nerves increases the risk of dry eye disease and infection. This study aims to evaluate the therapeutic potential of topical decorin to improve corneal nerve regeneration in a mouse model of sterile epithelial abrasion injury.

**Methods:**

Bilateral central corneal epithelial abrasions (2-mm, Alger Brush) were performed on young C57BL/6 J mice to remove the corneal sensory nerves. Decorin, or vehicle, was applied topically, three times per day for 1 week or every 2 h for 6 h. Spectral-domain optical coherence tomography was performed to measure the abrasion area and corneal thickness. Wholemount immunofluorescence staining was used to assess sensory nerve regeneration (β-tubulin III) and immune cell density (CD45, Iba1, CD11c). To investigate the specific role of dendritic cells (DCs), Cx3cr1^gfp/gfp^ mice, which spontaneously lack resident corneal epithelial DCs, were also investigated. The effect of prophylactic topical administration of recombinant human decorin (applied prior to the abrasion) was also investigated. Nerve tracing (NeuronJ software) was performed to compare recovery of basal nerve axons and superficial nerve terminals in the central and peripheral cornea.

**Results:**

At 6 h after injury, topical decorin application was associated with greater intraepithelial DC recruitment but no change in re-epithelialisation or corneal thickness, compared to the vehicle control. One week after injury, sub-basal nerve plexus and superficial nerve terminal density were significantly higher in the central cornea in the decorin-treated eyes. The density of corneal stromal macrophages in the decorin-treated eyes and their contralateral eyes was significantly lower compared to saline-treated corneas. No significant improvement in corneal nerve regeneration was observed in Cx3cr1^gfp/gfp^ mice treated with decorin.

**Conclusions:**

Decorin promotes corneal epithelial nerve regeneration after injury. The neuroregenerative effect of topical decorin was associated with a higher corneal DC density during the acute phase, and fewer macrophages at the study endpoint. The corneal neuroregenerative effects of decorin were absent in mice lacking intraepithelial DCs. Together, these findings support a role for decorin in DC-mediated neuroregeneration following corneal abrasion injury.

## Background

The cornea contains the highest density of sensory nerves and nociceptors compared to any other tissue in the body. The sensory nerve supply to the cornea originates from the ophthalmic division of the trigeminal nerve. Large nerve trunks enter the cornea at the peripheral limbus and form a network of stromal nerve trunks that turn anteriorly towards the epithelium at the ocular surface [1]. Unmyelinated nerve branches penetrate the epithelial basement membrane, divide further and run parallel along the basal epithelium towards the central cornea, forming the sub-basal nerve plexus (SBNP) [2]. Several nerve branches of the SBNP turn upward, penetrating vertically through the epithelium, and terminate just beneath the epithelial surface as superficial nerve terminals (SNT) [2]. This rich supply of unmyelinated peripheral nerves serves critical functions in maintaining homeostasis of the corneal epithelium and the ocular surface, orchestrating rapid responses to external stimuli with blinking, tear production and the release of numerous tropic substances, such as substance P, neurotransmitters and neuropeptides [3, 4]. A range of ocular surface conditions, including viral infections, chemical and physical burns, topical drug preservatives and corneal surgeries, can cause corneal neuropathy, which in turn can lead to chronic pain and dry eye disease [5, 6]. Systemic diseases such as diabetes mellitus can also negatively affect corneal nerve density and function [7, 8], thus compromising the integrity of the ocular surface.

Following experimental induction of a corneal epithelial abrasion in mice, recovery of the SBNP is typically incomplete by 4 weeks post-injury [9–11]. However, the apically located SNTs regenerate faster than the SBNP, providing evidence that these two inter-connected plexi have differential rates of recovery after injury [9]. Following stromal transection injury in mice, the density of the SBNP recovered to baseline levels after 6 weeks [12]. In humans, laser in situ keratomileusis (LASIK), the most common refractive surgery procedure, results in an 80% decrease in SBNP density at 5 days after surgery [13]. Restoration of the SBNP density to pre-operative levels takes at least 3 years [14, 15]. Furthermore, it is well established that during the first few months post-LASIK, about 30–40% of patients develop symptoms of dry eye disease [16] which has been linked to corneal epithelial sensory nerve damage [6, 17]. Current therapies, such as ocular lubricants, help alleviate ocular surface symptoms but do not treat the aetiology of the condition related to corneal denervation. Some factors, including nerve growth factor (NGF) and pigment epithelium-derived factor (PEDF), have been shown to be effective at accelerating corneal sensitivity after LASIK [18] and in promoting corneal nerve regeneration in preclinical models of nerve injury post-viral infection [19] respectively.

Although the cornea is avascular, several studies have characterised the resident immune cells that exist in the corneal stroma (mostly macrophages, with a small population of CD11c^+^ dendritic cells (DCs) [20, 21]) and epithelium (almost exclusively CD11c^+^ DCs [22–25]). In addition to co-ordinating innate inflammatory responses after injury [26, 27], these cells also contribute to maintenance of nerves and lymphatic vessels [23, 28]. The macrophages, distributed predominantly in the anterior stroma, appear to make physical contacts with corneal nerve trunks in the peripheral cornea [29]. The intraepithelial CD11c^+^ DCs decrease in density from the peripheral limbus to the central cornea [20, 21, 30]. Using an inducible model of DC depletion (CD11c-DTR mice), Gao et al. showed that resident corneal DCs contribute to corneal nerve density during homeostasis and after sterile injury [23] via production of ciliary neurotrophic nerve factor (CNTF). In the healthy human cornea, the density of corneal nerves was positively correlated with resident corneal DC density, suggesting an interaction between corneal nerves and immune cells even in steady state conditions [31, 32].

Decorin is a small, leucine-rich proteoglycan that exists in most connective tissues, including in the trabecular meshwork, sclera and cornea of the eye [33]. Although decorin is considered a structural component of the extracellular matrix, it mediates a diverse range of cellular processes including collagen fibrillogenesis, wound healing, fibrosis, neovascularisation and inflammation [34]. In the cornea, impaired expression of decorin, caused by a frameshift mutation, is responsible for opacification in congenital stromal corneal dystrophy [35]. It has also been reported that decorin plays anti-fibrotic roles in animal models of proliferative vitreoretinopathy and *Pseudomonas* keratitis [36, 37]. In a rabbit model of corneal neovascularisation (CNV), decorin gene therapy delivered with adeno-associated virus serotype 5 decreased CNV, through a mechanism involving downregulation of vascular endothelial growth factor expression [38]. Notably, in the central nervous system, decorin has been reported to promote nerve axon growth following spinal cord injury in vivo, and in the cultured dorsal root ganglia [39–41]. In addition, decorin has been reported to regulate inflammation in in vitro studies, including rescuing macrophages from apoptosis and enhancing their activation by blocking endogenously produced transforming growth factor beta (TGF-β) [42, 43]. However, the effect of decorin on corneal nerve regeneration has not been previously investigated. Therefore, the aim of this study was to assess the effects of topical human recombinant decorin on corneal nerve regeneration in a well-established murine model of epithelial abrasion injury. In addition, changes to corneal immune cells were investigated to explore the potential mechanism(s) involved in the neuroregenerative response.

## Materials and methods

### Animals

Wild-type female C57BL6 mice (6–8 weeks old) were purchased from the Animal Resources Centre, Murdoch, Western Australia, and housed in a specific pathogen-free environment at the Florey Institute of Neuroscience and Mental Health. Cx3cr1-deficient (Cx3cr1 ^gfp/gfp^) mice that spontaneously lack resident corneal epithelial DCs [44] and CD11c-eYFP reporter mice that harbour transcriptional control of the mouse integrin alpha X (CD11c) promoter, thus labelling resident corneal DCs, were included in the study. All animals were treated in accordance with the ARVO Statement for the Use of Animals in Ophthalmic and Vision Research, and all procedures were approved by the Animal Ethics Committee at the Florey Institute of Neuroscience and Mental Health (18-094-UM).

### Spectral domain optical coherence tomography

In vivo spectral domain optical coherence tomography (SD-OCT) imaging was performed to measure the corneal wound sizes at baseline (time 0 hours [h]) and to measure corneal epithelial and stromal thickness at the experimental endpoint. Corneal epithelial thickness was quantified as a safety measure, to exclude the possibility of any unexpected effects of decorin on corneal epithelial wound healing and to verify that decorin did not influence epithelial cell proliferation or oedema. Anesthetised mice were placed on the animal imaging mount and rodent alignment stage (AIM-RAS) attached to the SD-OCT imaging device (Bioptigen Envisu R2200 VHR; Bioptigen, Inc., Durham, NC, USA). Volumetric 3 × 3-mm rectangular scans of the central cornea (1000 A-scans/200 B-scans) were captured using an 18-mm telecentric lens at baseline and immediately after the abrasion (0 h), 6 h and 1-week post corneal injury. Central corneal thickness (CCT) was measured as previously described [45] using ImageJ software (http://imagej.nih.gov/ij/; provided in the public domain by the National Institutes of Health, Bethesda, MD, USA). En face images were used to measure the size of the epithelial abrasion area using a freehand trace tool in ImageJ.

### Corneal abrasion injury

Mice were anesthetised with an intraperitoneal injection of a ketamine (80 mg/kg) and xylazine (10 mg/kg) diluted with saline. A corneal abrasion injury was performed on both eyes of each animal as described previously [9]. In brief, an approximately 2-mm diameter circular area of the central corneal epithelium was demarcated using a sterile 2-mm trephine, then debrided using an ophthalmic burr (0.5 mm, Algerbrush II; Alger Equipment Co., Lago Vista, TX, USA). Immediately following debridement, a 2-μl drop of sterile saline was applied to each eye to prevent corneal drying.

### Preparation of decorin, decorin fluid gel and fluid gel eye drops

Decorin fluid gels and fluid gels (without decorin) were produced using low acyl gellan gum (Kelco gel CG LA, Azelis, UK) as previously described [46]. The fluid gels were used to provide localised drug delivery and improved retention on the ocular surface [37]. The fluid gels are optically transparent and are naturally removed through shear forces generated with blinking over a duration of 4–6 h. The decorin fluid gel formulation was prepared by adding human recombinant decorin (4.76 mg/ml; Galacorin^TM^, Catalent Pharma Solutions, NJ, USA) in phosphate-buffered saline (PBS) and aqueous sodium chloride (0.2 M), with the final concentrations of 0.9% (w/v) gellan, 0.24 mg/ml decorin and 10 mM NaCl. The fluid gel (without decorin) was adjusted to a final composition of 0.9% (w/v) gellan and 10 mM NaCl.

### Topical treatment

**Fig. 1:**
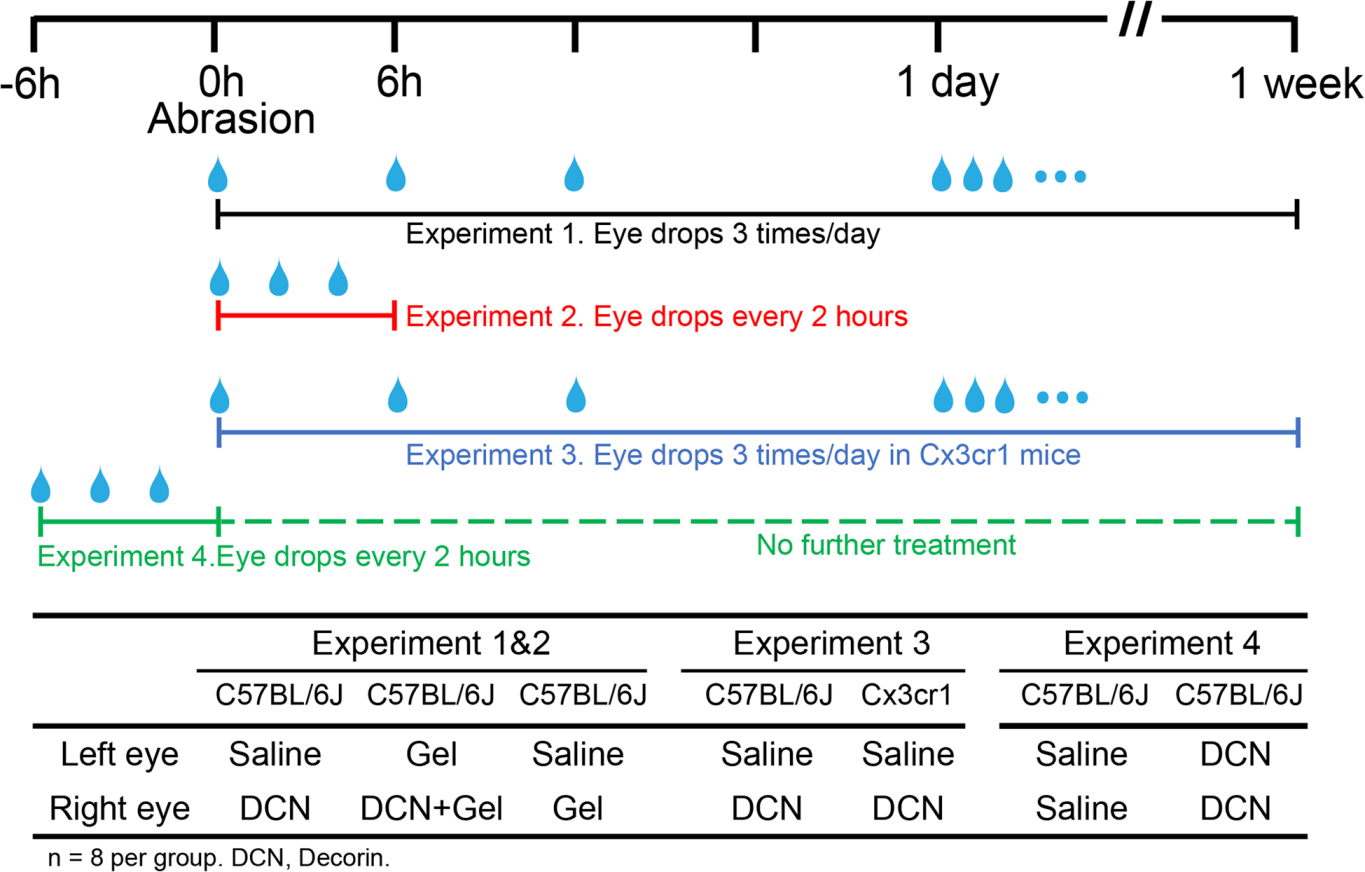
Schematic diagram of the study timepoints and specific treatments for the four complementary experimental components. Experiment 1: After corneal abrasion, wild-type mice received eye drops 3 times per day for 1 week; Experiment 2: After corneal abrasion, wild-type mice received eye drops every 2 h (0 h, 2 h, 4 h) and were then euthanised at 6 h; Experiment 3: After corneal abrasion, Cx3cr1^gfp/gfp^ and wild-type mice received eye drops 3 times per day for 1 week; Experiment 4: wild-type mice received eye drops every 2 h (-6 h, -4 h, -2 h) before abrasion (0 h), and were then housed for 1 week without further treatment

Mice received eye drops (5 μl of either decorin (4.76 mg/ml in PBS; Galacorin^TM^, Catalent, USA), saline, decorin fluid gel or fluid gel only) either three times per day for 1 week (1-week timepoint; see Experiment 1, Fig. 1) or three times administered at two hourly intervals over a 6-h period (see Experiment 2, Fig. 1). Mice were held for 1 min after each eye drop to allow the eye drops to distribute across the ocular surface. A small group of CD11c-eYFP mice (*n* = 5) was included in Experiment 2 to verify that intraepithelial CD45^+^ DCs expressed CD11c^+^ (see Supplemental file Fig. S1).

### Role of DCs in corneal nerve regeneration

Cx3cr1 ^gfp/gfp^ mice lack resident corneal epithelial DCs [44]. To investigate the potential role of resident and early infiltrating DCs in corneal nerve regeneration, a group of Cx3cr1^gfp/gfp^ mice (*n* = 8) and wild-type C57BL/6 J mice (*n* = 8) were examined 1 week after a ~ 2-mm corneal epithelial injury and daily application of either decorin or saline eye drops (see Experiment 3, Fig. 1).

To determine whether topical application of decorin on an intact cornea could induce corneal DC infiltration, wild-type C57BL6 mice (*n* = 3) received topical decorin every 2 h for 6 h before euthanasia. To determine whether preconditioning with decorin could impart a protective effect prior to corneal epithelial injury, pre-treatment with decorin eye drops or vehicle was applied 3 times at 2 hourly intervals at -6 h, -4 h, -2 h pre-injury (0 h). Mice were euthanised after 1 week, and the extent of corneal nerve regeneration was quantified (see Experiment 4, Fig. 1).

### Wholemount immunofluorescence

Mice were euthanised, and dissected corneas were fixed in 100% methanol for 1 h at 4 °C and then washed in PBS. Corneal flat mounts were incubated in 20 mM ethylenediaminetetraacetic acid for 60 min at 37 °C and blocked with 3% bovine serum and 0.3% Triton X-100 in PBS for 60 min at room temperature. For immunostaining, tissues were incubated overnight at 4 °C with primary antibody rabbit anti-β tubulin III (1:500; #T2200, Sigma, St Louis, MO, USA) and rat anti-CD45 (1:500; #550539, BD Biosciences, Franklin Lakes, NJ, USA) or rabbit anti-Iba1 (1:500; #019-19741, Wako, Osaka, Japan) and rat anti-Ki67 (1:500; #14-5698-80, eBiosciences, Carlsbad, CA, USA). Afterwards, tissue flat mounts were washed with PBS before incubation with the secondary antibodies, goat anti-rabbit Alexa Fluor 647 (1:500; #A21244, ThermoFisher Scientific, Carlsbad, CA, USA) and goat anti-rat Alexa Cy3 (1:500; #A10522, ThermoFisher Scientific, Carlsbad, CA, USA) and Hoechst (1:1000; Sigma, St Louis, MO, USA) for 120 min at room temperature. Immunostained samples were then washed and mounted onto glass slides with aqueous mounting medium and coverslipped for imaging.

### Corneal nerve and immune cell image acquisition and analysis

Corneal whole mounts were imaged using a confocal microscope with a × 40 objective lens (Confocal Laser Scanning Microscopy SP8; Leica Microsystems, Buffalo Grove, IL, USA). Three non-overlapping z-series (z-step size 1 μm, image size 290 × 290 μm) were captured from the central (within central 1.5 mm of the cornea) and peripheral cornea (between 2 mm and 2.5 mm from the centre of the cornea) respectively. Separate z-stacks of the SNTs and SBNP were created by generating z-projections of the superficial and basal epithelial layers [9, 47]. To compare the innate inflammatory response between groups, z-stack images of the anterior corneal stroma (5 μm directly below the basal epithelium) were created for analysing neutrophils (CD45^+^ Iba1^−^ with a distinct polymorphonuclear appearance) and macrophages (CD45^+^ Iba1^+^). For analysis of DC density, distribution and dendritic field area, one image was collected from the central cornea and three images from the peripheral cornea using an Olympus BX51 microscope with a × 10 objective lens (900 μm × 600 μm area).

### Image analysis

All images were analysed by a masked observer. The sum length of SNTs and SBNP was quantified using the NeuronJ plugin in ImageJ software for manual nerve tracing as previously described [9, 47, 48]. The density of macrophages, neutrophils and DCs was counted manually, and density of Ki67^+ve^ epithelial cells was analysed using automated threshold counting in ImageJ. Macrophages were identified by CD45^+^ Iba1^+^ staining and location within the stroma. DCs were identified by being CD45^+^ and displaying a distinct dendriform morphology and location within the epithelium.

### Statistical analyses

For the mice who received different treatments in each eye, the data analysis was performed by fitting a linear mixed-effects model using restricted maximum likelihood (REML) and Kenward-Roger tests for fixed effects. The model included fixed effects of decorin, fluid gel and contralateral effects along with the two-way interactions of decorin and gel, and gel and contralateral effect. The mouse model was included as a random effect to account for correlation between the eyes of a particular animal. After fitting the model, post hoc tests were performed to examine the three main effects including decorin, fluid gel and any potential ‘contralateral eye effect’ from the decorin intervention. For the experiments only involving application of decorin and saline, a Wilcoxon Signed Ranks test or Wilcoxon Rank-Sum test was performed. All statistical analyses were performed in Stata software (version 14.2; StataCorp LLC, College Station, TX, USA). A *p* < 0.05 was considered statistically significant. All summary data are shown as mean ± SD.

## Results

### Corneal neuroregenerative and inflammatory effects of decorin at 1 week

**Fig. 2:**
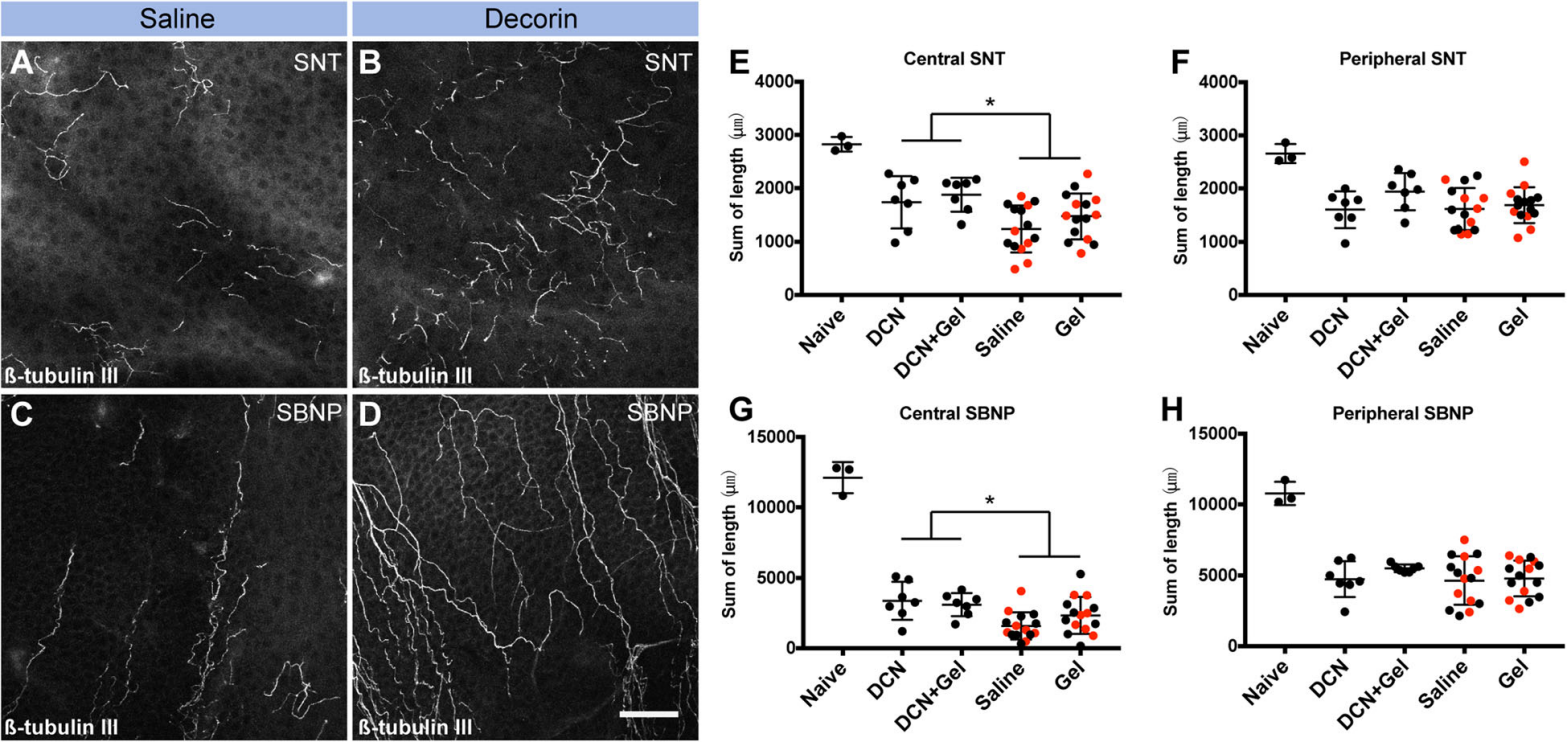
Experiment 1: Corneal nerve regeneration after 1 week of topical decorin treatment, dosed 3 times/day. **a**–**d** Representative confocal images of the SNTs and SBNP in the central cornea after saline **(a**, **c**) and decorin (**b**, **d**) topical treatment. **e**–**f** Quantification of the sum length of SNTs in the central and peripheral cornea. **g**–**h** Sum length of the SBNP in the central and peripheral cornea. Summary data are shown as mean ± SD. Scale bar in **d** (50 μm) applies to all images. Red symbols in **e**–**h** represent the contralateral eye of the decorin-treated eye. Asterisk indicates a statistically significant difference between eyes with and without decorin treatment. *P* values for each of the inter-group comparisons are provided in Table 1. Legend: DCN, decorin; Gel, fluid gel

For all experiments, the abrasion injury was photographed for every animal to ensure similar wound sizes were generated in all mice. In Experiment 1, the baseline corneal epithelial injury was 2.83 ± 0.50 mm^2^ (mean ± SD), with no inter-group difference in injury size before the topical treatments (see Supplemental file 2). Table 1 summarises results from the mixed-effects statistical models on corneal nerve regeneration and immune cell density, including the effects of decorin, fluid gel, the potential contralateral eye effect and their interactions. The eyes treated with decorin showed greater corneal nerve regeneration in the central cornea compared to those without (SNT 1806 ± 402 vs 1355 ± 443 μm, *p* = 0.027; SBNP 3208 ± 1085 vs 1963 ± 1196 μm, *p* = 0.006). This effect was not observed in the peripheral cornea, which was the area adjacent to the 2-mm wound margin (Fig. 2). There was no difference between fluid gel + decorin and decorin alone observed for all measurements, indicating that the decorin fluid gel did not provide any additional improvement to the effect of decorin alone.

**Table 1:** Experiment 1: Comparison of corneal nerve parameters and immune cell densities in eyes with different topical treatments after 1 week

	Main effects						Interactions	
	Decorin		Fluid gel		Contralateral eye effect		Decorin # Gel	Gel # Contralateral
	Contrast (95% CI)	*p* value	Contrast (95% CI)	*p* value	Contrast (95% CI)	*p* value	*p* value	*p* value
Central cornea								
SNT (μm)	402.1 (49.0, 755.2)	**0.027**	277.0 (− 125.3, 679.3)	0.166	− 103.2 (− 456.4, 249.9)	0.552	0.840	0.249
SBNP (μm)	1350.0 (427.3, 2272.7)	**0.006**	152.9 (− 871.6, 1177.3)	0.758	159.6 (− 763.1, 1082.3)	0.725	0.155	0.687
DCs (cells/mm^2^)	0.3 (− 1.9, 2.6)	0.781	− 1.8 (− 4.2, 0.6)	0.138	0.0 (− 2.3, 2.2)	0.968	0.301	0.469
Macrophages (cells/mm^2^)	− 72.6 (− 109.2, − 36.1)	**< 0.001**	16.1 (− 25.7, 58.0)	0.430	− 52.0 (− 88.5, − 15.4)	**0.007**	0.925	0.787
Ki67+ (cells/mm^2^)	265.1 (− 126.9, 657.0)	0.177	− 22.6 (− 446.2, 401.0)	0.912	− 41.2 (− 433.2, 350.7)	0.831	0.103	0.557
Peripheral cornea								
SNT (μm)	109.9 (− 181.4, 401.2)	0.446	221.5 (− 102.0, 545.0)	0.168	− 26.5 (− 317.8, 264.8)	0.853	0.253	0.801
SBNP (μm)	475.4 (− 706.4, 1657.2)	0.414	413.9 (− 958.6, 1786.5)	0.535	149.5 (− 1032.3, 1331.3)	0.796	0.530	0.851
DCs (cells/mm^2^)	0.0 (− 3.9, 4.0)	0.984	0.9 (− 3.5, 5.3)	0.670	− 1.3 (− 5.2, 2.7)	0.523	0.108	0.713
Macrophages (cells/mm^2^)	− 45.7 (− 70.9, − 20.4)	**0.001**	14.7 (− 13.5, 43.0)	0.288	− 42.9 (− 68.1, − 17.6)	**0.002**	0.855	0.695
Ki67+ (cells/mm^2^)	63.6 (− 253.2, 380.5)	0.684	− 150.3 (− 495.8, 195.2)	0.373	83.2 (− 233.7, 400.0)	0.595	0.224	0.095
Corneal thickness at apex								
Epithelium (μm)	0.7 (− 1.0, 2.4)	0.400	1.3 (− 0.6, 3.3)	0.170	1.1 (− 0.6, 2.9)	0.193	0.910	0.727
Stroma (μm)	0.2 (− 2.6, 3.1)	0.868	− 1.8 (− 5.1, 1.5)	0.259	1.7 (− 1.2, 4.6)	0.231	0.189	0.054

**Fig. 3:**
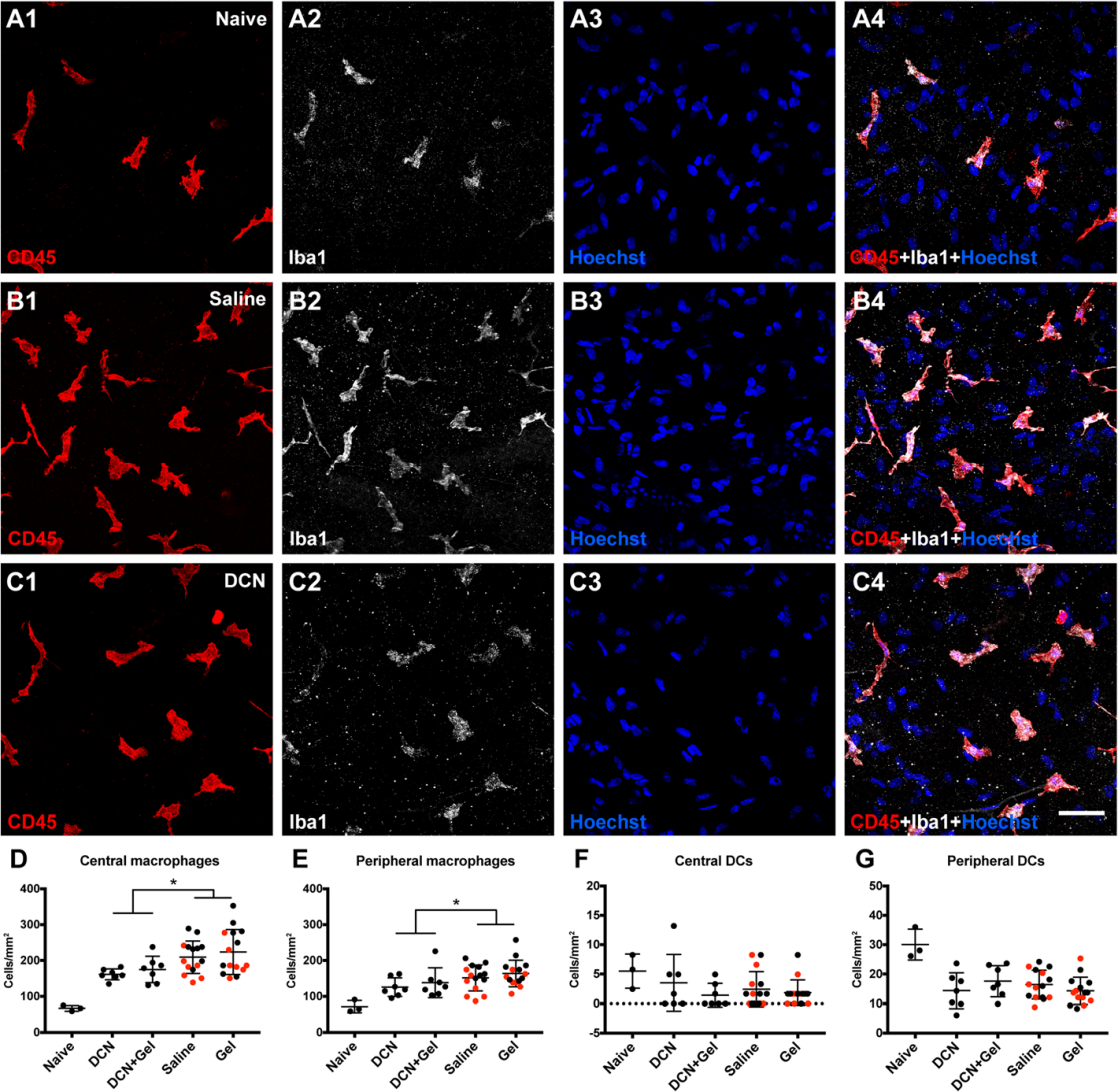
Experiment 1: Changes to corneal immune cells after 1 week of topical treatment, dosed 3 times/day. **a**–**c** Representative confocal images of the anterior stromal CD45^+^ Iba1^+^ macrophages in the central cornea of naïve (**a**1–**a**4), saline-treated (**b**1–**b**4) or decorin-treated eye (**c**1–**c**4). Scale bar in **c**4 (50 μm) applies to all images**. d**, **e** Density of anterior stromal macrophages in the central and peripheral cornea. **f**, **g** Density of epithelial DCs in the central and peripheral cornea. Summary data are shown as mean ± SD. Each data point represents a single cornea. Red symbols represent the contralateral eye of the decorin-treated eye. Asterisk indicates a statistically significant difference between eyes with and without decorin treatment. *P* values for each of the inter-group comparisons are provided in Table 1. Legend: DCN, decorin; Gel, fluid gel

Statistics are derived from a mixed-effects model. Data are also shown in Figs. 2, 3 and Supplemental file Fig. S3. *P* value indicates the significance of the contrast between the eyes with or without the intervened effect (decorin, fluid gel and etc.)

*CI* confidence interval, *DCs* dendritic cells, *SBNP* Sub-basal nerve plexus, *SNT* superficial nerve terminals

Macrophage density in the anterior stroma and DC density in the epithelium were quantified in the central and peripheral cornea (Fig. 3). At 1 week, the density of macrophages was significantly lower in decorin-treated eyes at both corneal eccentricities (Fig. 3a and b; *p* < 0.001 and *p* = 0.001, respectively). Interestingly, the saline-treated corneas contralateral to decorin-treated corneas had a lower density of macrophages in both the central and peripheral cornea (*p* = 0.007 and 0.002 respectively) compared to corneas that received saline or fluid gel only, suggesting a contralateral eye effect of decorin on macrophage density. At the 1-week timepoint, there was no difference in DC density in corneas treated with topical decorin, decorin fluid gel or fluid gel (Fig. 3c and d).

To verify that topical application of decorin did not induce any unexpected corneal inflammation, epithelial thickness (see Supplemental file 3A-C, in vivo SD-OCT) and cell proliferation (Supplemental file 3D-F, ex vivo Ki67 staining) were assessed. There were no differences in epithelial cell proliferation or corneal thickness after applying decorin and/or the fluid gel for 1 week.

### Effect of topical decorin on corneal immune cells and re-epithelialisation at 6 h

In order to explore whether the increased nerve regeneration in decorin-treated corneas was due to faster re-epithelialisation after injury and if there were any differences in the number or distribution of inflammatory cells during the acute phase of wound healing, a similar experiment was conducted, but tissues examined after 6 h (Experiment 2). The results are summarised in Table 2. Topical application of decorin and/or the fluid gel did not alter the extent of re-epithelialisation relative to the saline control (Supplemental file 4A-C). Eyes treated with the fluid gel had a higher stromal thickness at 6 h compared to those without (*p* = 0.002) (Supplemental file 4D).

**Table 2:** Experiment 2: Comparison of immune cell densities in eyes with different topical treatments after 6 h (i.e., 3× doses, 2 h apart)

	Main effects						Interactions	
	Decorin		Fluid gel		Contralateral eye effect		Decorin # Gel	Gel # Contralateral
	Contrast (95% CI)	*p* value	Contrast (95% CI)	*p* value	Contrast (95% CI)	*p* value	*p* value	*p* value
Re-epithelialisation (%)	2.4 (− 3.1, 8.0)	0.377	− 3.9 (− 9.9, 2.1)	0.192	0.1 (− 5.5, 5.7)	0.977	0.982	0.878
Stromal thickness (μm)	− 0.8 (− 9.8, 8.1)	0.854	16.5 (7.1, 26.0)	**0.002**	2.5 (− 6.4, 11.3)	0.573	0.204	0.153
Peripheral cornea								
DCs (cells/mm^2^)	15.7 (7.6, 23.8)	**< 0.001**	− 4.6 (− 13.0, 3.7)	0.261	3.4 (− 4.7, 11.5)	0.400	0.200	0.248
DC area (μm^2^/cell)	119.3 (− 68.8, 307.4)	0.205	− 172.6 (− 369.7, 24.5)	0.083	137.1 (− 51.0, 325.2)	0.147	0.460	0.302
Macrophages (cells/mm^2^)	− 6.9 (− 23.4, 9.5)	0.396	− 0.2 (− 18.0, 17.5)	0.977	− 10.4 (− 26.9, 6.1)	0.206	0.196	0.832
Neutrophils (cells/mm^2^)	− 144.5 (− 343.9, 54.9)	0.150	− 135.4 (− 342.3, 71.5)	0.188	− 281.1 (− 480.5, − 81.8)	**0.007**	0.071	0.317

**Fig. 4:**
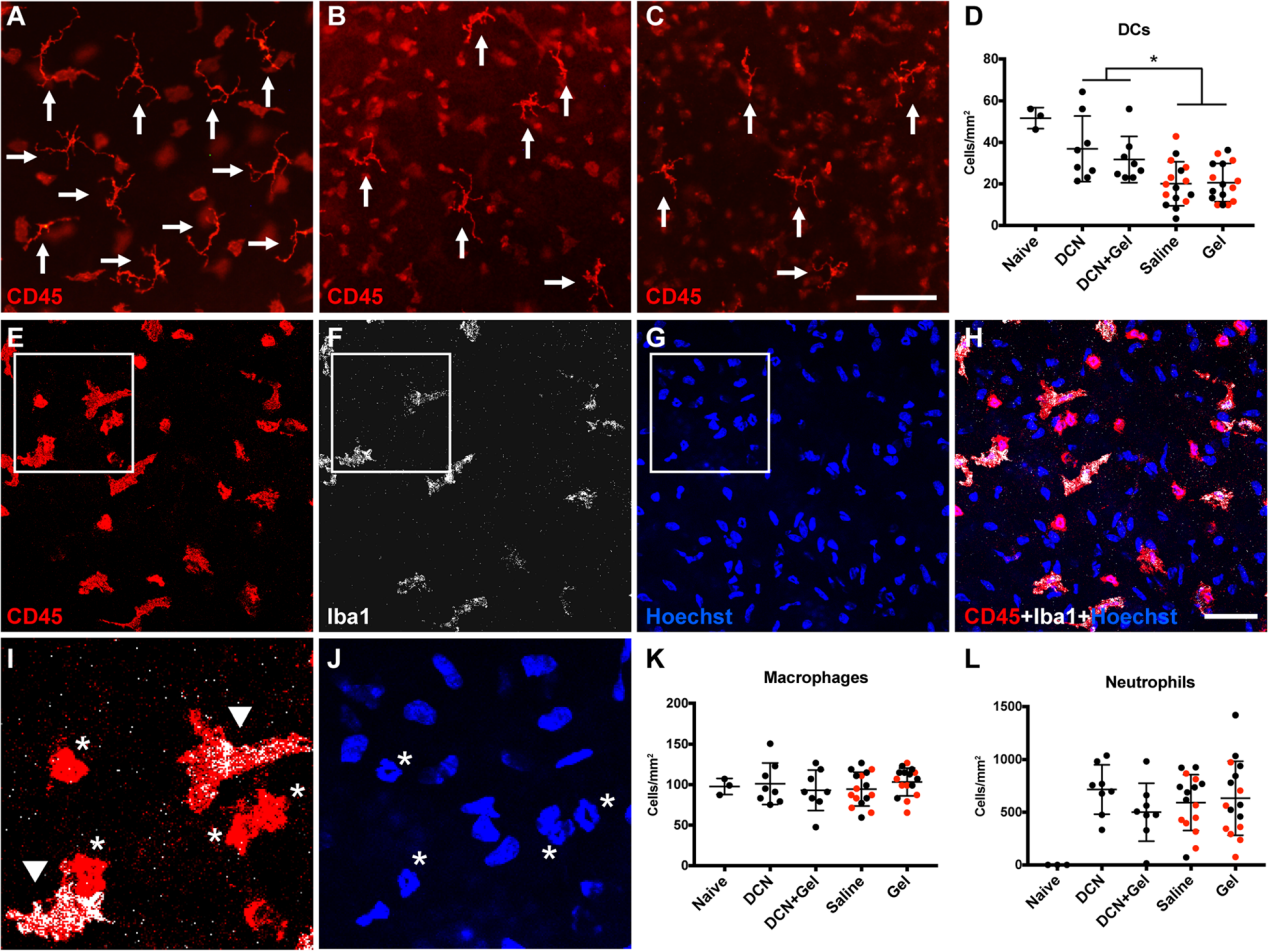
Experiment 2: Changes to immune cells after 6-hours of topical treatment (i.e., 3 × doses, 2 hours apart). **a–c** Representative immunofluorescence images from the peripheral corneas of naïve (**a**), decorin-treated injured (**b**) and saline-treated injured (**c**) eyes. Arrows indicate intraepithelial CD45^+^ DCs. Scale bar in **c** (100 μm) applies to **a**–**c**. **d** Density of DCs in the peripheral corneal epithelium after 6 h of treatment. **e–h** Representative confocal images of CD45^+^ Iba1^+^ cells in the peripheral corneal stroma. Scale bar in **h** (50 μm) applies to **e**–**h**. **i** A higher magnification and merged image of the boxed area in **e** and **f**. Arrowheads indicate CD45^+^ Iba1^+^ stromal macrophages; asterisks indicate CD45^+^ Iba1^−^ neutrophils. **j** A higher magnification image of the boxed area in **g**. Asterisks indicate distinct polymorphonuclear appearance of neutrophil nuclei. **k**, **l** Density of macrophages and neutrophils in the peripheral corneal stroma after 6 h of treatment. Summary data are shown as mean ± SD. Each data point represents one cornea. Red symbols in **f**–**i** represent the contralateral eye of the decorin-treated eye. Asterisk indicates a statistically significant difference between eyes with and without decorin treatment. *P* values for each of the inter-group comparisons are provided in Table 2. Legend: DCN, decorin; Gel, fluid gel

Statistics are derived from mixed-effects models. Data are also shown in Fig. 4 and Supplemental file Fig. S4. *P* value indicates the significance of the contrast between the eyes with or without the intervened effect (decorin, fluid gel and etc.)

*CI* confidence interval, *DC* dendritic cell

DC density was significantly higher (*p* < 0.001) in the peripheral cornea of the eyes treated with decorin (Fig. 4a–c) compared to those without decorin treatment, while no difference was observed in the field area of DCs (Fig. 4d). Compared to the finding after 1 week of decorin treatment where macrophage numbers were reduced, there was no effect on macrophage density with decorin at 6-h post-injury (Fig. 4e–k). The density of infiltrated neutrophils at 6-h post-injury showed no difference between the eyes with different treatments (Fig. 4l).

### Corneal neuroregenerative effect of topical decorin in Cx3cr1^gfp/gfp^ mice

**Fig. 5:**
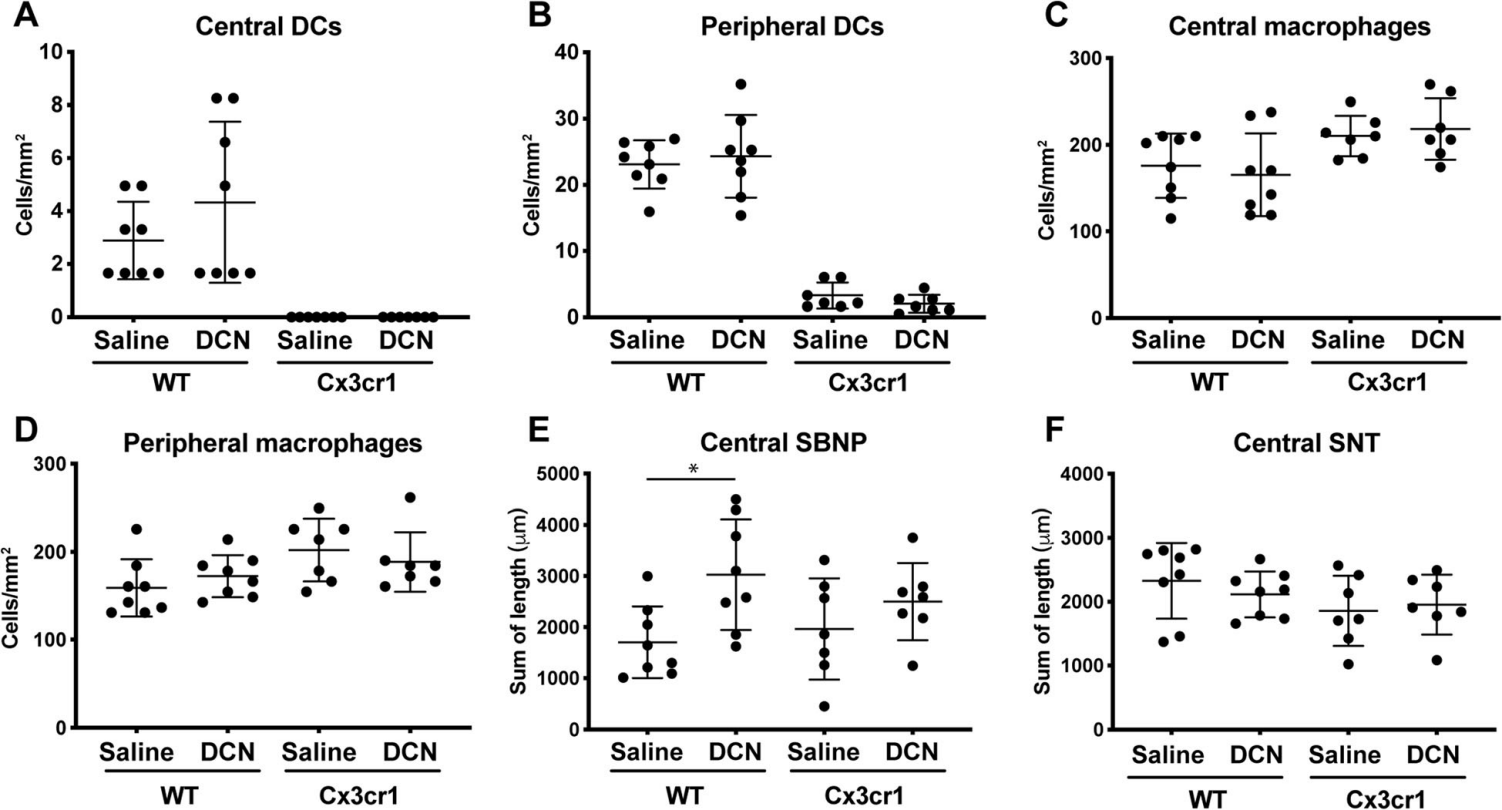
Experiment 3: Corneal immune cell density and nerve regeneration in Cx3cr1^gfp/gfp^ mice after 1-week of topical treatment with saline or decorin (DCN). **a–b** Density of epithelial DCs in the central and peripheral cornea. **c**–**d** Density of anterior stromal macrophages in the central and peripheral cornea. **e**–**f** Quantification of the sum length of the SNTs and SBNP in the central cornea respectively. Summary data are shown as mean ± SD. Asterisk indicates significant difference between saline-treated and decorin-treated eyes. Each data point represents 1 cornea. Legend: DC, dendritic cell; DCN, decorin; WT, wild-type; *n* = 8 WT, *n* = 7 Cx3cr1^gfp/gfp^ mice

Based on previous reports of the role of resident corneal epithelial DCs in epithelial nerve recovery after injury [23], Cx3cr1^gfp/gfp^ mice were used to investigate whether the decorin-mediated nerve regeneration would occur in the absence of resident epithelial DCs (Experiment 3). As expected, Cx3cr1^gfp/gfp^ mice showed fewer DCs in the central and peripheral cornea compared to WT mice after 1 week (Fig. 5a and b). Consistent with the previous experiments, the density of macrophages was similar between paired eyes receiving decorin and saline, due to the contralateral effect of decorin on macrophage density (Fig. 5c). Notably, the decorin-treated eye in WT C57BL/6 J mice showed improved SBNP regeneration (*p* = 0.039), but this effect was not apparent in Cx3cr1^gfp/gfp^ mice (Fig. 5d). There was no significant inter-group difference for SNT regeneration (Fig. 5e).

### Corneal neuroregenerative and immunomodulatory effects of topical decorin treatment administered 6 h prior to injury

To determine if topical application of decorin on the intact cornea could act as a chemoattractant, DCs were quantified in the corneal epithelium, 6 h after the instillation of eye drops (3 doses, every 2 h) on healthy, uninjured eyes. A higher density of DCs in the peripheral, but not the central, cornea was observed (Supplemental file 5A&B). To investigate the possibility that pre-conditioning decorin treatment could induce a higher density of DCs and promote corneal nerve regeneration, decorin-treated mice received topical decorin prior to epithelial abrasion and were housed for 1-week after injury without any further topical treatments. No significant inter-group difference in the extent of corneal nerve regeneration (in the SNTs or SBNP) or immune cell density (DCs and macrophages) was observed at 1 week (Supplemental file 5C-F), suggesting decorin pre-injury short-pulse pre-conditioning is not effective at promoting corneal nerve recovery after epithelial injury.

## Discussion

Corneal sensory nerves are vital for maintaining epithelial integrity [49]. It follows that corneal nerve dysfunction is the pathophysiologic basis of a variety of ocular surface diseases. During the corneal nerve regenerative process, the eye is vulnerable to dry eye disease and epithelial erosions, which can cause ocular pain and discomfort. Clinical studies show that it can take several years for corneal nerves to completely regenerate after surgical-induced ablation [14, 15, 50], potentially leading to secondary ocular surface conditions, such as dry eye disease and neurotrophic keratopathy [17, 49, 51]. Despite the clinical importance of treatments to promote corneal nerve regeneration, there are relatively few therapeutic approaches. Some clinical studies have reported corneal neuroregenerative effects with topical or oral omega-3 fatty acid supplementation in individuals with damaged corneal nerves [52, 53]. However, there are currently few therapies available to improve nerve regeneration after injury. In the present study, we examined whether topical decorin influenced corneal nerve regeneration after sterile injury. Using a mouse model of central corneal epithelial abrasion, which removes all sensory nerves at the site of injury, we showed that corneal nerve fibre length was higher after 1 week of topical decorin therapy relative to inactive topical treatment (i.e., saline or fluid gel). Furthermore, topical decorin treatment was associated with a higher density of intraepithelial DCs during the acute phase (6 h post injury), and a lower density of stromal macrophages at 1 week.

Esquenazi et al. reported that recombinant mature NGF treatment improved corneal nerve regeneration at 8 weeks after photorefractive keratectomy (PRK) in a rabbit model [54]. Interestingly, their study also showed increased epithelial cell proliferation after NGF treatment, providing evidence of an interaction between corneal nerves and epithelial cells. The epithelium can provide anatomical structural support for corneal sensory nerves and release neurotrophic mediators that induce neurite survival [55]. In the present study, we did not find any difference in corneal re-epithelialisation either by in vivo SD-OCT imaging or epithelial cell proliferation (by Ki-67 staining) at 1 week after topical decorin treatment. These results suggest that decorin may exert its effect on corneal nerve regeneration by other mechanisms.

Decorin is a known inhibitor of TGF-β, binding to and neutralising its biological activities and regulating TGF-β signalling pathways by interacting with low density lipoprotein receptor-related protein-1 (LRP-1) [56]. Moreover, decorin can interact with many immune-related proteins including Toll-like receptors that are involved in the regulation of inflammation [57] and Class A scavenger receptors that are specifically expressed on macrophages [58]. Interestingly, mounting evidence highlights that there is a strong interaction between corneal inflammation and nerve regeneration [59, 60]. In a mouse model of corneal lamellar transection surgery, Namavari et al. [61] reported that Sema7A, an axon guidance factor, can promote corneal nerve regeneration and increase inflammatory cell influx (CD11b^+^ cells and CD3^+^ lymphocytes) into the cornea, suggesting Sema7A may act as a neurotrophic factor by regulating inflammatory processes. However, this study only assessed the total length of nerves in the epithelium and stroma. It is unclear if the SBNP and SNTs recovered at similar rates or if any topographical differences in nerve recovery in response to Sema7A existed. In contrast, our study finds that corneal stromal macrophages, but not epithelial DCs, have a lower density after 1 week of topical decorin treatment; these findings suggest that decorin may exert immunomodulatory effects in the cornea post-injury. Similar to our findings, He et al. observed a faster resolution of CD4+ and CD11b + cell inflammation in a rabbit model of corneal herpes simplex virus type-1 (HSV-1) infection, after topical treatment with PEDF plus docosahexaenoic acid (DHA) for 12 weeks, which was accompanied by improved corneal nerve regeneration [19]. The interactions between macrophages and injured peripheral nerves have been well studied in other tissues [62, 63], with macrophages responsible for the phagocytosis of myelin debris. However, most corneal nerves are unmyelinated or thinly myelinated [2]. Although a previous study has demonstrated a physical association between corneal stromal nerve trunks and resident corneal macrophages in uninjured eyes [29], further studies are required to investigate how stromal macrophages may participate in the corneal epithelial nerve regeneration process.

In order to explore the acute phase inflammatory response after exposure of the intact ocular surface to decorin, we measured corneal immune cell density after 6 h and found a significantly higher density of DCs in the corneas treated with topical decorin relative to controls. A recent study has shown a mechanistic association between corneal intraepithelial DCs and sensory nerves, with DCs proposed to produce neurotrophic factors, such as CNTF, which promote corneal nerve regeneration after injury [23]. Moreover, Colorado et al. reported that corneal DC density in healthy eyes was positively correlated with corneal nerve fibre area and branch density, assessed using in vivo confocal microscopy (IVCM) [32]. In addition, a clinical study of patients with infectious keratitis reported a strong negative relationship between corneal nerve density and the number of putative DCs in the central cornea [59].

The contralateral eye effects of immunological and neural pathology have been well described in human and animal studies. In humans, the uninfected cornea contralateral to an infectious keratitis has been shown to have a heightened DC density, and the cells demonstrate an altered morphology [64]. In mice, substance P was released in the eye contralateral to the eye with a neural injury, involving a sympathetic response [65]. In a mouse model, Jiao et al. observed enlarged corneal DC morphology in the eye contralateral to a unilateral corneal epithelial injury [24]. Although no contralateral effect was observed with topical decorin for corneal SBNP regeneration and DC density, the macrophages in the contralateral eye of the decorin-treated eye had a lower density at 1 week compared to the non-decorin-treated group. A possible explanation is that decorin may directly impact stromal cytokine production, sequestering growth factors and downregulating chemokines that recruit macrophages. Another explanation is that the improved corneal nerve regeneration locally restores epithelial homeostasis and thus reduces the epithelial stress that recruits stromal macrophages. This may also explain why the change to macrophage density was only observed at 1 week, when there was a significant improvement in nerve regeneration after topical decorin treatment, supporting our hypothesis that the change to DCs at early stages of corneal healing may be primarily responsible for the improved nerve regeneration. These findings justify future studies to measure the effect of decorin on cytokine and chemokine expression in the healthy and injured cornea.

The optically transparent fluid gel used in these studies is formulated to transition between liquid and solid states allowing for higher rates of ocular retention whilst providing a lubricating effect. Decorin fluid gels are scheduled to enter into Phase IIa clinical trials in 2020 (EudraCT registration: 2017-000389-32) [37]. Although the fluid gel was introduced to prolong the retention of decorin on the ocular surface in this study, we did not observe any difference in the corneal neuroregenerative effects of decorin fluid gel when compared to decorin alone. One potential explanation is that topical decorin was applied three times per day for 1 week or every 2 h during the early stage (6 h) observation window, which could be sufficiently frequent to retain an effective concentration of decorin on a debrided ocular surface. Application in humans though might require the use of fluid gels to reduce the need for such frequent topical administration; this would also be expected to have the added potential effect of lubricating the eye to reduce irritation.

To further investigate the role of DCs in corneal nerve regeneration, we applied decorin eye drops following corneal injury in Cx3cr1^gfp/gfp^ mice that spontaneously lack resident corneal epithelial DCs. In WT mice, the decorin-treated eye showed greater regeneration in the SBNP than the contralateral saline-treated eye in C57BL/6 J mice. This effect was not observed in Cx3cr1^gfp/gfp^ mice. These findings suggest that the absence of DCs might abrogate decorin-mediated corneal nerve regeneration. Gao et al. have demonstrated that a local depletion of DCs can delay the corneal nerve regeneration in a mouse model of corneal epithelial debridement, though it was unclear if this delayed nerve regeneration was isolated to the SBNP or SNTs [23]. Interestingly, another study by the same laboratory reported that intraepithelial DCs produce CNTF that can promote axon regeneration, providing a potential mechanism for DC-dependent corneal nerve regeneration after injury [66]. A recent study by our group, reported that eyes treated with a decorin fluid gel had improved corneal re-epithelialisation 16 days after the induction of *Pseudomonas* keratitis [37]. However, it is difficult to compare epithelial wound healing between studies, as the infectious model of *Pseudomonas* keratitis is more severe than the sterile injury.

In addition, when comparing the sum length of SNTs between decorin- and saline-treated corneas in WT and Cx3cr1^gfp/gfp^, there was no significant difference in either of the groups. This result may initially appear inconsistent with the results of our first experiment. However, post hoc review of the initial corneal abrasion areas revealed a significant difference between the two experiments, with the second study having larger wound sizes (see Supplemental file 6A). Notably, according to our epithelial wound area data (see Supplemental file 6B) and other previous studies [67, 68], the area of regenerated epithelium was positively correlated with the size of the initial corneal injury. Therefore, it is possible that compared to Experiment 1 (WT mice with 1-week DCN), the relatively larger injured area in Experiment 3 (WT and Cx3cr1^gfp/gfp^ mice with 1-week DCN) may have initiated a larger re-epithelialised area. We propose that the regenerated epithelium may have provided more anatomical structural support for SNTs to sprout in the acute phase of regeneration. Moreover, it has been shown that corneal epithelial cells can phagocytose axonal debris after nerve injury [69]. The removal of axonal debris plays an important role in the initiation of the corneal sensory nerve regeneration [70, 71]. Therefore, the regenerated corneal epithelial cells may provide a favourable microenvironment for corneal reinnervation after nerve injury.

Pre-injury, short-duration pre-treatment with decorin did not show any benefits to corneal nerve recovery after epithelial injury; however, we did observe a higher density of DCs in the peripheral epithelium of the intact cornea. The small sample size (*n* = 3) of this proof-of-concept study is acknowledged as a limitation, but the significant finding justified further investigations to explore how decorin interacts with DCs in the intact cornea.

## Conclusions

Our data provide evidence that topical decorin treatment can promote corneal nerve regeneration, predominantly in the central SBNP, in part by increasing the number of DCs in the acute phase post-injury. The bilateral effects of unilateral decorin instillation could be triggered by the corneal nerve regeneration, or as a result of decorin altering the cytokine milieu of the corneal stroma. It would be interesting to define the cytokine, chemokine and neuropeptide responses in corneas (both direct and contralateral) after treatment with decorin, as well as to determine the optimal dose of decorin (to inform clinical translation) and to determine whether the regenerated nerves provide functional improvements.

## Supplementary information

**Additional file 1: Figure S1**. Experiment 2: CD45^+^ dendritic cells (DCs) after 6-hours of topical treatment (i.e., 3 × doses, 2 hours apart) in CD11c-eYFP mice. **(A)** CD45^+^ CD11c^+^ DCs in peripheral cornea after decorin treatment. **(B)** CD45^+^ CD11c^+^ DCs in peripheral cornea after saline treatment. Scale bar (50 μm) applies to all images. **Figure S2**. Experiment 1: Initial corneal abrasion area at baseline (Time 0h). **Figure S3.** Experiment 1: Corneal thickness and epithelial cell proliferation after 1-week of topical treatment, dosed 3 times/day. **(A)** Representative SD-OCT images of the anterior segment from naïve mice. Blue curved line represents the area of central cornea and blue dashed line represents peripheral cornea. Scale bar is 200 μm. **(B)** A higher magnification image of the boxed area in A. Orange and green double arrows indicate epithelial and stromal thickness respectively. **(C)** Corneal epithelial and stromal thickness. There were no significant inter-group differences. **(D)** Representative *en face* confocal image of Ki67 staining in the central cornea after 1-week treatment of decorin. Scale bar is 50 μm. **(E-F)** Density of proliferative epithelial cells in the central and peripheral cornea. Summary data are shown as mean ± SD. Each data point represents one cornea. Red symbols represent the contralateral eye of the decorin-treated eye. Legend: DCN, decorin; Gel, fluid gel. **Figure S4.** Experiment 2: Corneal re-epithelialisation and stromal thickness after 6-hours of topical treatment (i.e., 3 × doses, 2 hours apart). **(A)** Representative *en face* OCT image at baseline (0h after abrasion). **(B)** Representative *en face* OCT image after 6-hours of treatment. Red dashed lines in panels A and B indicate the margin of the injured epithelium. Scale bar in B is 0.5 mm. **(C)** Percentage of re-epithelialised corneal area after 6-hours of treatment. **(D)** Corneal stromal thicknesses after 6-hours of treatment. Red symbols in panels C and D represent the contralateral eye of the decorin-treated eye. Summary data are shown as mean ± SD. Each data point represents one cornea. P-values for each of the inter-group comparisons are provided in Table 2. Legend: DCN, decorin; Gel, fluid gel. **Figure S5.** Experiment 4: Effect of topical decorin applied before injury (DCN) on corneal immune cells and nerve regeneration. **(A-B)** Density of DCs in the central and peripheral corneal epithelium after topical application of prophylactic decorin on intact corneas. **(C-D)** Sum length of the SNTs and SBNP in the central cornea, at 1 week after prophylactic application of decorin. **(E-F)** Density of DCs in the peripheral epithelium and macrophages in the central stroma, at 1 week after prophylactic application of topical decorin. Summary data are shown as mean ± SD. * indicates a statistically significant difference between saline-treated and decorin-treated eyes. Each data point represents one cornea. Legend: DC, dendritic cell; DCN, decorin; SBNP, sub-basal nerve plexus; SNT, superficial nerve terminal; WT, wild-type. **Figure S6 (A)** Comparison of initial abrasion area between Experiment 1 and 3. **(B)** Relationship between the initial abrasion area and the re-epithelialised area at 6h.

## Data Availability

The datasets used and/or analysed during the current study are available from the corresponding author on reasonable request.

## Abbreviations

CCT: Central corneal thickness
CNTF: Ciliary neurotrophic nerve factor
CNV: Corneal neovascularisation
DC: Dendritic cell
NGF: Nerve growth factor
SD-OCT: Spectral domain optical coherence tomography
PEDF: Pigment epithelium-derived factor
SBNP: Sub-basal nerve plexus
SNT: Superficial nerve terminal
TGF-β: Transforming growth factor beta
