# Supplementary material for: The neuroregenerative effects of topical decorin on the injured mouse cornea
Source: J Neuroinflammation. 2020 May 4;17:142. doi: 10.1186/s12974-020-01812-6 (PMC7199348; doi:10.1186/s12974-020-01812-6)
Supplement: Supplementary file 1 — Additional file 1: Figure S1. Experiment 2: CD45+ dendritic cells (DCs) after 6-hours of topical treatment (i.e., 3 × doses, 2 hours apart) in CD11c-eYFP mice. (A) CD45+ CD11c+ DCs in peripheral cornea after decorin treatment. (B) CD45+ CD11c+ DCs in peripheral cornea after saline treatment. Scale bar (50 μm) applies to all images. Figure S2. Experiment 1: Initial corneal abrasion area at baseline (Time 0h). Figure S3. Experiment 1: Corneal thickness and epithelial cell proliferation after 1-week of topical treatment, dosed 3 times/day. (A) Representative SD-OCT images of the anterior segment from naïve mice. Blue curved line represents the area of central cornea and blue dashed line represents peripheral cornea. Scale bar is 200 μm. (B) A higher magnification image of the boxed area in A. Orange and green double arrows indicate epithelial and stromal thickness respectively. (C) Corneal epithelial and stromal thickness. There were no significant inter-group differences. (D) Representative en face confocal image of Ki67 staining in the central cornea after 1-week treatment of decorin. Scale bar is 50 μm. (E-F) Density of proliferative epithelial cells in the central and peripheral cornea. Summary data are shown as mean ± SD. Each data point represents one cornea. Red symbols represent the contralateral eye of the decorin-treated eye. Legend: DCN, decorin; Gel, fluid gel. Figure S4. Experiment 2: Corneal re-epithelialisation and stromal thickness after 6-hours of topical treatment (i.e., 3 × doses, 2 hours apart). (A) Representative en face OCT image at baseline (0h after abrasion). (B) Representative en face OCT image after 6-hours of treatment. Red dashed lines in panels A and B indicate the margin of the injured epithelium. Scale bar in B is 0.5 mm. (C) Percentage of re-epithelialised corneal area after 6-hours of treatment. (D) Corneal stromal thicknesses after 6-hours of treatment. Red symbols in panels C and D represent the contralateral eye of the decorin-treat [file 12974_2020_1812_MOESM1_ESM.docx]

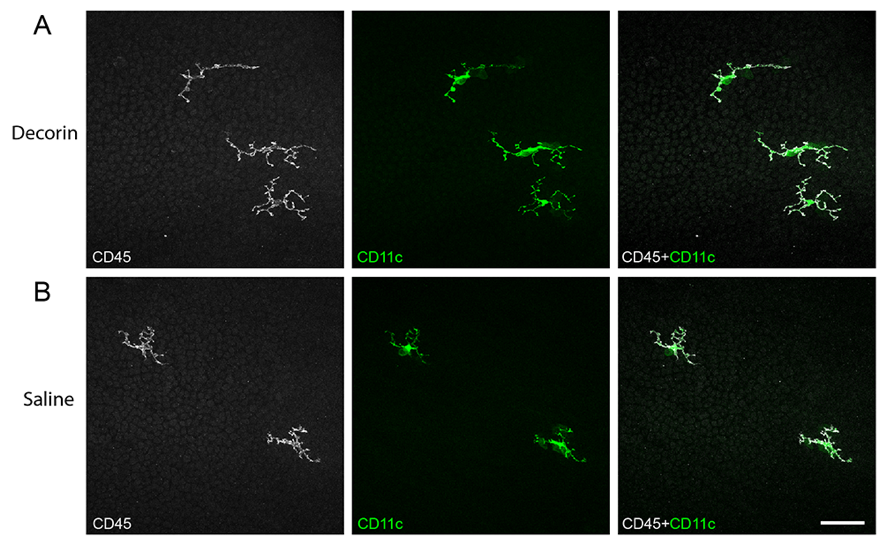


**Figure S1.** Experiment 2: CD45^+^ dendritic cells (DCs) after 6-hours of topical treatment (i.e., 3 × doses, 2 hours apart) in CD11c-eYFP mice. **(A)** CD45^+^ CD11c^+^ DCs in peripheral cornea after decorin treatment. **(B)** CD45^+^ CD11c^+^ DCs in peripheral cornea after saline treatment. Scale bar (50 µm) applies to all images.


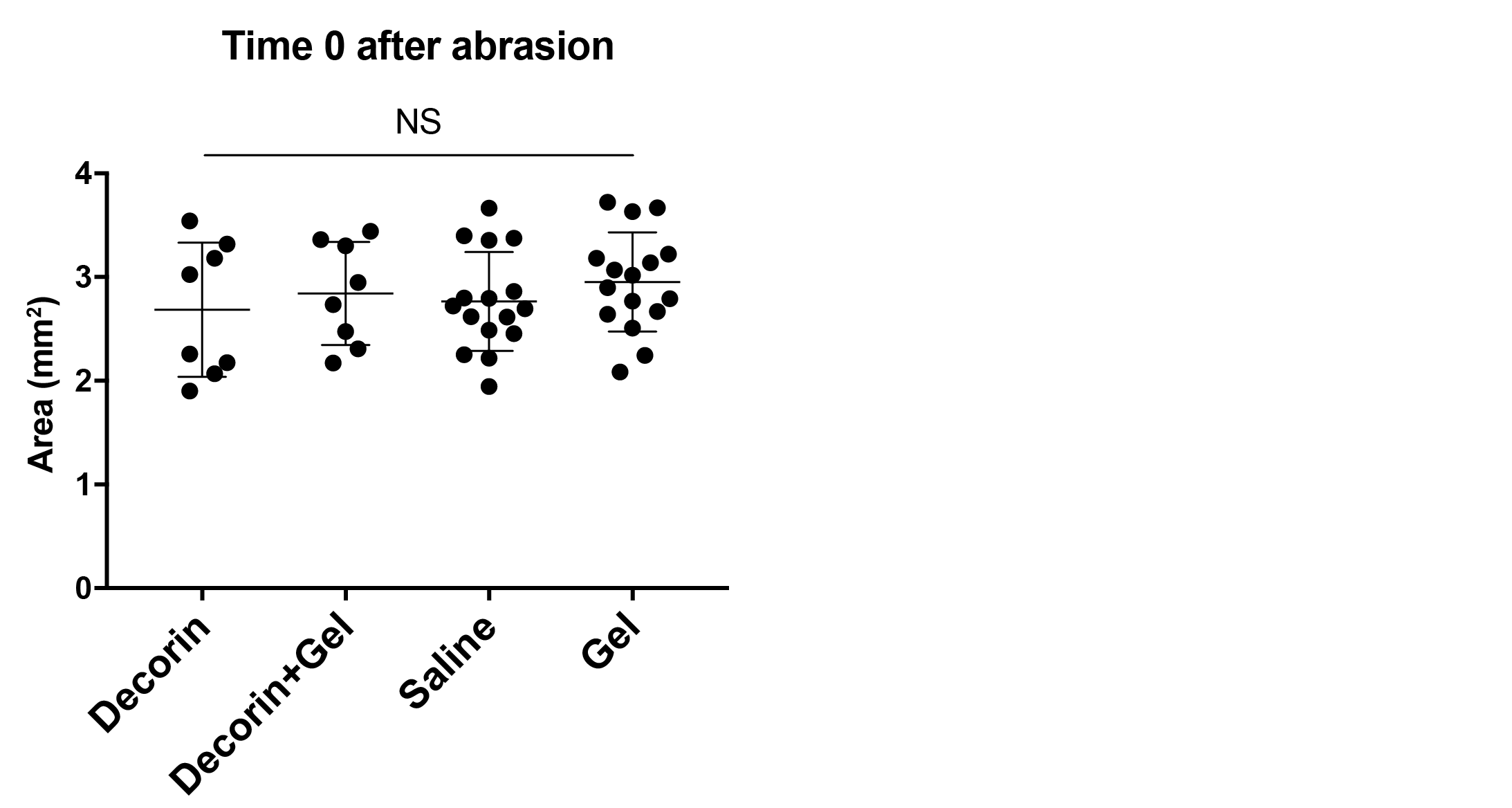


**Figure S2**. Experiment 1: Initial corneal abrasion area at baseline (Time 0h).


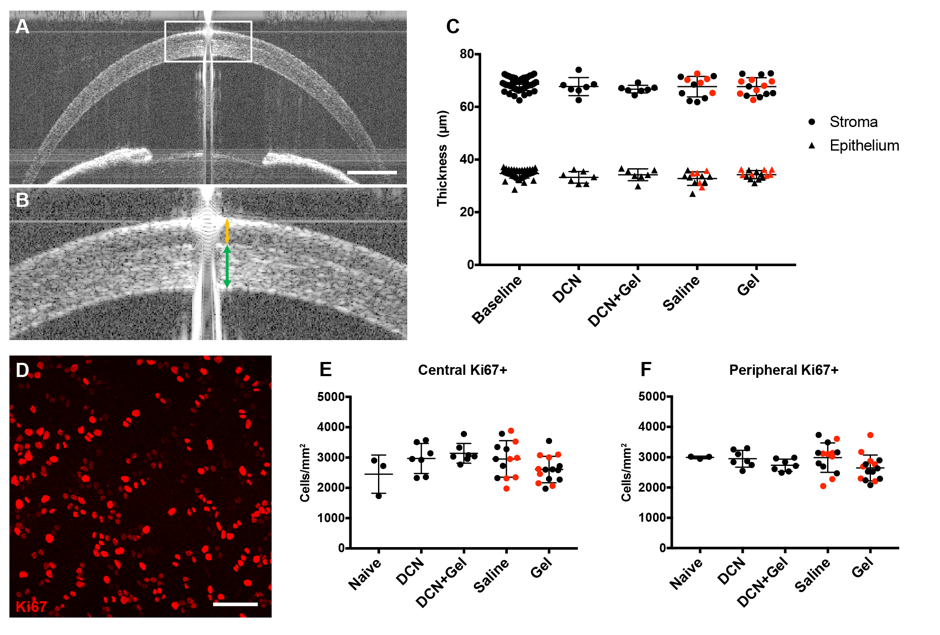


**Figure S3.** Experiment 1: Corneal thickness and epithelial cell proliferation after 1-week of topical treatment, dosed 3 times/day. **(A)** Representative SD-OCT images of the anterior segment from naïve mice. Blue curved line represents the area of central cornea and blue dashed line represents peripheral cornea. Scale bar is 200 µm. **(B)** A higher magnification image of the boxed area in A. Orange and green double arrows indicate epithelial and stromal thickness respectively. **(C)** Corneal epithelial and stromal thickness. There were no significant inter-group differences. **(D)** Representative *en face* confocal image of Ki67 staining in the central cornea after 1-week treatment of decorin. Scale bar is 50 µm. **(E-F)** Density of proliferative epithelial cells in the central and peripheral cornea. Summary data are shown as mean ± SD. Each data point represents one cornea. Red symbols represent the contralateral eye of the decorin-treated eye. Legend: DCN, decorin; Gel, fluid gel.


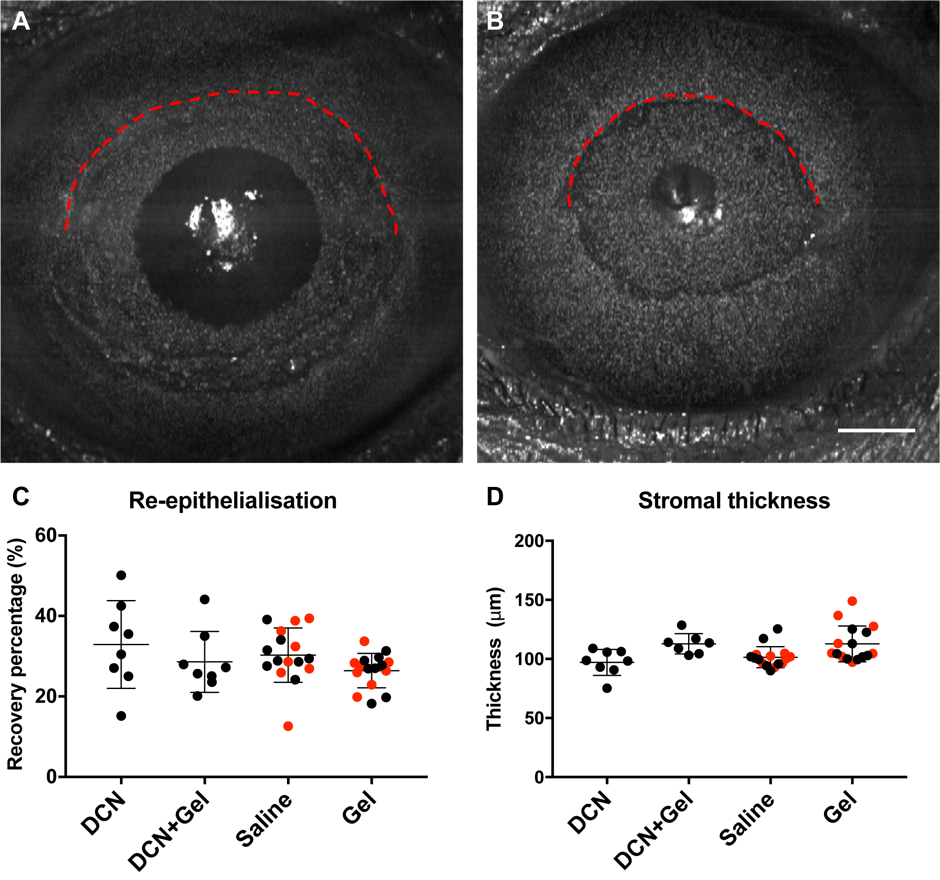


**Figure S4.** Experiment 2: Corneal re-epithelialisation and stromal thickness after 6-hours of topical treatment (i.e., 3 × doses, 2 hours apart). **(A)** Representative *en face* OCT image at baseline (0h after abrasion). **(B)** Representative *en face* OCT image after 6-hours of treatment. Red dashed lines in panels A and B indicate the margin of the injured epithelium. Scale bar in B is 0.5 mm. **(C)** Percentage of re-epithelialised corneal area after 6-hours of treatment. **(D)** Corneal stromal thicknesses after 6-hours of treatment. Red symbols in panels C and D represent the contralateral eye of the decorin-treated eye. Summary data are shown as mean ± SD. Each data point represents one cornea. P-values for each of the inter-group comparisons are provided in Table 2. Legend: DCN, decorin; Gel, fluid gel.


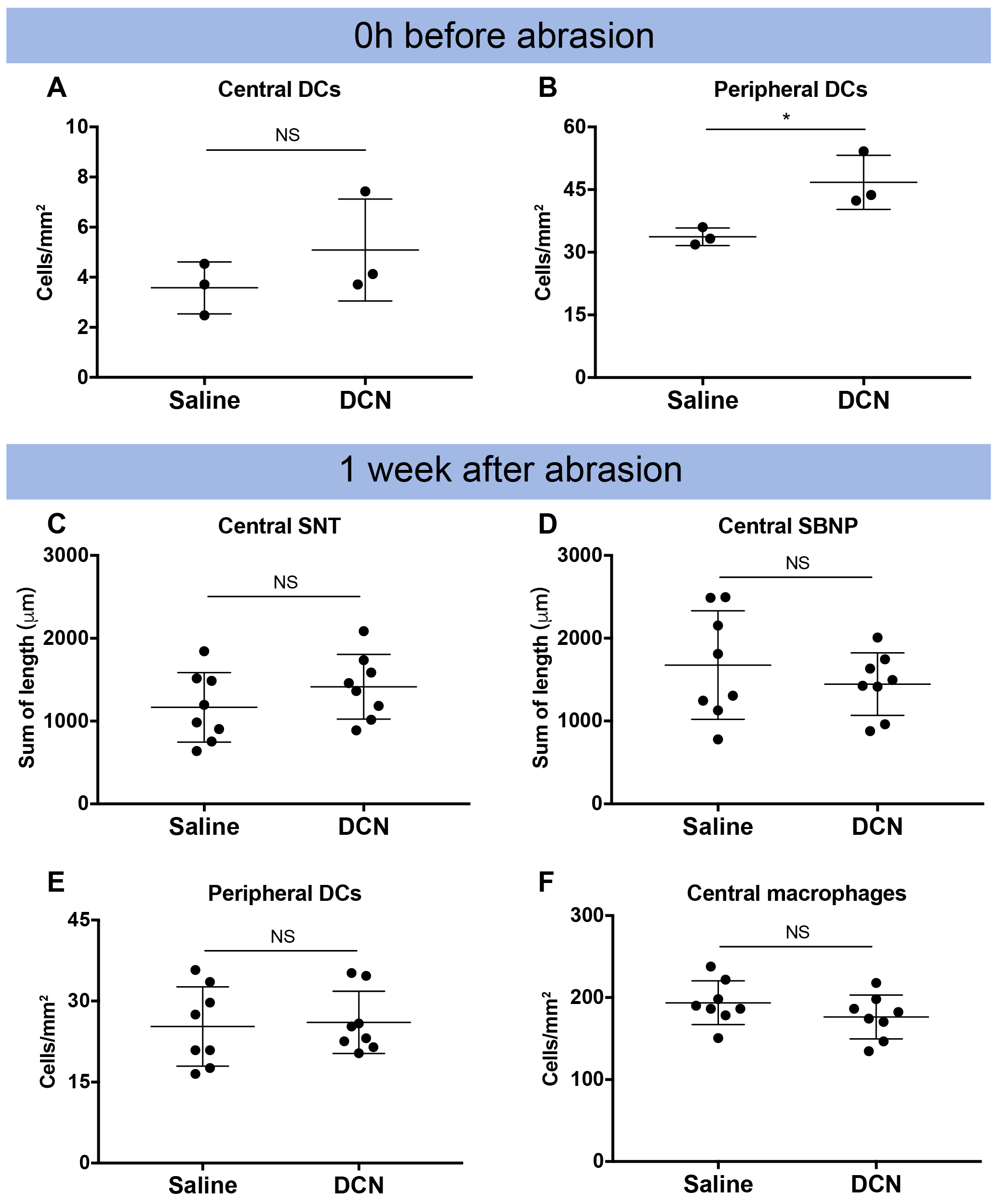


**Figure S5.** Experiment 4: Effect of topical decorin applied before injury (DCN) on corneal immune cells and nerve regeneration. **(A-B)** Density of DCs in the central and peripheral corneal epithelium after topical application of prophylactic decorin on intact corneas. **(C-D)** Sum length of the SNTs and SBNP in the central cornea, at 1 week after prophylactic application of decorin. **(E-F)** Density of DCs in the peripheral epithelium and macrophages in the central stroma, at 1 week after prophylactic application of topical decorin. Summary data are shown as mean ± SD. * indicates a statistically significant difference between saline-treated and decorin-treated eyes. Each data point represents one cornea. Legend: DC, dendritic cell; DCN, decorin; SBNP, sub-basal nerve plexus; SNT, superficial nerve terminal; WT, wild-type.


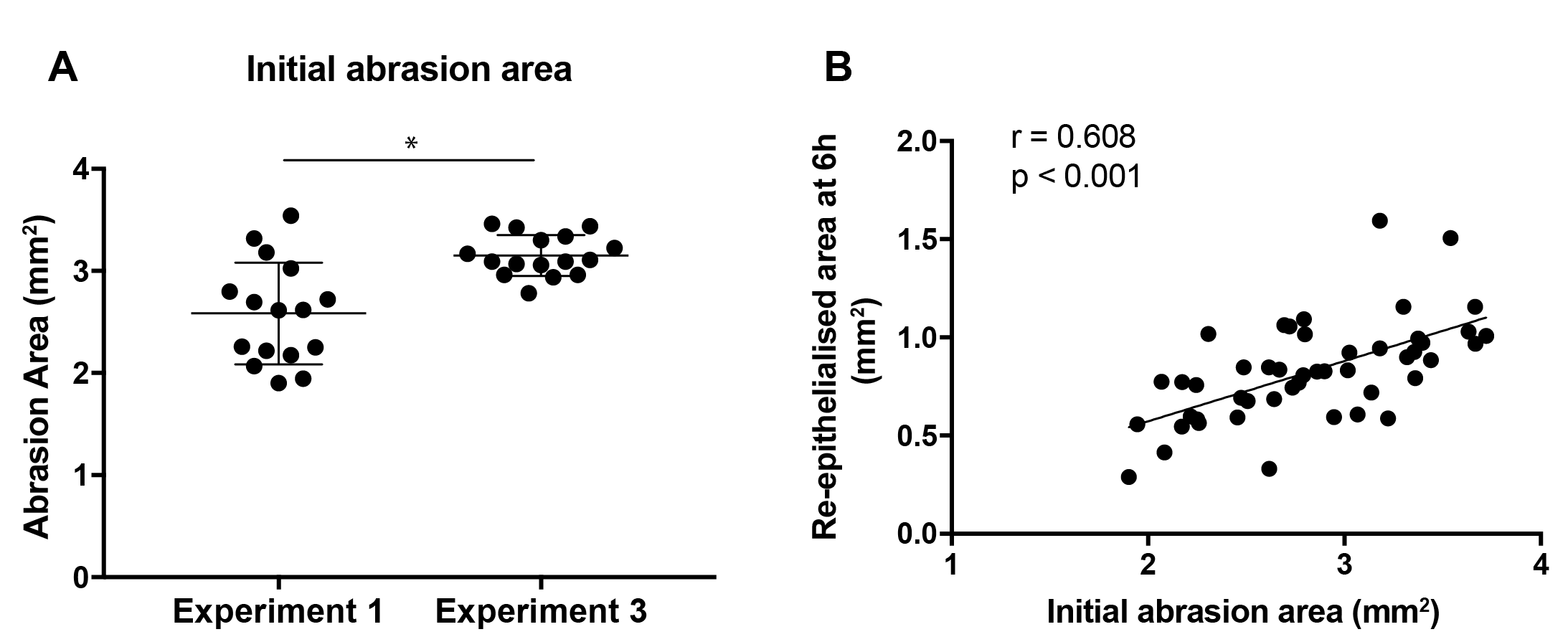


**Figure S6.** **(A)** Comparison of initial abrasion area between Experiment 1 and 3. **(B)** Relationship between the initial abrasion area and the re-epithelialised area at 6h.
